# Evaluation of an AI-Powered Lung Nodule Algorithm for Detection and 3D Segmentation of Primary Lung Tumors

**DOI:** 10.1155/2019/1545747

**Published:** 2019-07-01

**Authors:** Thomas Weikert, Tugba Akinci D'Antonoli, Jens Bremerich, Bram Stieltjes, Gregor Sommer, Alexander W. Sauter

**Affiliations:** Department of Radiology, University Hospital Basel, University of Basel, Petersgraben 4, 4031 Basel, Switzerland

## Abstract

Automated detection and segmentation is a prerequisite for the deployment of image-based secondary analyses, especially for lung tumors. However, currently only applications for lung nodules ≤3 cm exist. Therefore, we tested the performance of a fully automated AI-based lung nodule algorithm for detection and 3D segmentation of primary lung tumors in the context of tumor staging using the CT component of FDG-PET/CT and including all T-categories (T1–T4). FDG-PET/CTs of 320 patients with histologically confirmed lung cancer performed between 01/2010 and 06/2016 were selected. First, the main primary lung tumor within each scan was manually segmented using the CT component of the PET/CTs as reference. Second, the CT series were transferred to a platform with AI-based algorithms trained on chest CTs for detection and segmentation of lung nodules. Detection and segmentation performance were analyzed. Factors influencing detection rates were explored with binominal logistic regression and radiomic analysis. We also processed 94 PET/CTs negative for pulmonary nodules to investigate frequency and reasons of false-positive findings. The ratio of detected tumors was best in the T1-category (90.4%) and decreased continuously: T2 (70.8%), T3 (29.4%), and T4 (8.8%). Tumor contact with the pleura was a strong predictor of misdetection. Segmentation performance was excellent for T1 tumors (*r* = 0.908, *p* < 0.001) and tumors without pleural contact (*r* = 0.971, *p* < 0.001). Volumes of larger tumors were systematically underestimated. There were 0.41 false-positive findings per exam. The algorithm tested facilitates a reliable detection and 3D segmentation of T1/T2 lung tumors on FDG-PET/CTs. The detection and segmentation of more advanced lung tumors is currently imprecise due to the conception of the algorithm for lung nodules <3 cm. Future efforts should therefore focus on this collective to facilitate segmentation of all tumor types and sizes to bridge the gap between CAD applications for screening and staging of lung cancer.

## 1. Introduction

Failure to detect lung cancer on imaging studies is a very common reason for malpractice suits [1]. The reasons for misdiagnosis are multilayered and include recognition error and satisfaction of search [2]. Strategies for the reduction of observer errors are therefore of great importance and computer-aided detection (CAD) of pulmonary nodules has gained increasing interest in this context [3]. Most recently, conventional CAD solutions that require visual confirmation to reduce false-positive calls [4] are being challenged by deep learning algorithms that have an inherent advantage of automatic feature exploitation [3].

The diagnostic task of imaging in lung cancer, however, does not end with tumor detection. Tumor staging using 18F-fluorodeoxyglucose- (FDG-) PET/CT as the standard of care forms an integral part of the clinical diagnostic workup of patients with lung cancer [5]. The recent revision on the T-categories for the 8^th^ edition of the TNM lung cancer classification emphasized that from 1 to 5 cm, each cm separates lesions of significantly different prognosis [6]. However, the implicit assumption that tumors are spherical and consequently proportional changes of tumor diameter and parallel changes in tumor volume is particularly disrupted for advanced tumors [7]. This clearly underlines the need for accurate tumor segmentation and precise tumor volumetry, particularly when it comes to therapy response monitoring [7], radiation treatment planning [8], radiomics [9], and other new developments in the framework of personalized medicine.

Sexauer et al. have shown that manual annotation and segmentation of lung tumors is feasible, but tumor stage and lesion size and count correlate significantly with segmentation time [10]. Algorithms for automatic pulmonary nodule detection and segmentation are currently under development but are commonly trained and validated based on intraparenchymal lesions which are less than 3 cm in size. Therefore, it is unclear how pulmonary masses beyond this diameter and with nonspherical shape will be treated by these algorithms. Moreover, the vast majority of CAD systems have been evaluated on chest CTs that have been acquired in deep-inspiration breath-hold technique [11–21]. So far, only few CAD applications were tested for PET/CT and that only for nodules smaller than 3 cm [22, 23].

It was thus the aim of this study to evaluate the performance of a fully automated computer-assisted detection and 3D segmentation algorithm that was initially designed for lung nodule detection and segmentation in the context of tumor staging. This was done using the CT component of FDG-PET/CT studies of a patient cohort with histologically proven primary lung tumors from all T-categories.

## 2. Materials and Methods

This study was conducted under the provisions of the appropriate Swiss regional ethics committee (*Ethikkommission Nordwest-und Zentralschweiz*).

### 2.1. Case Selection

**Figure 1:**
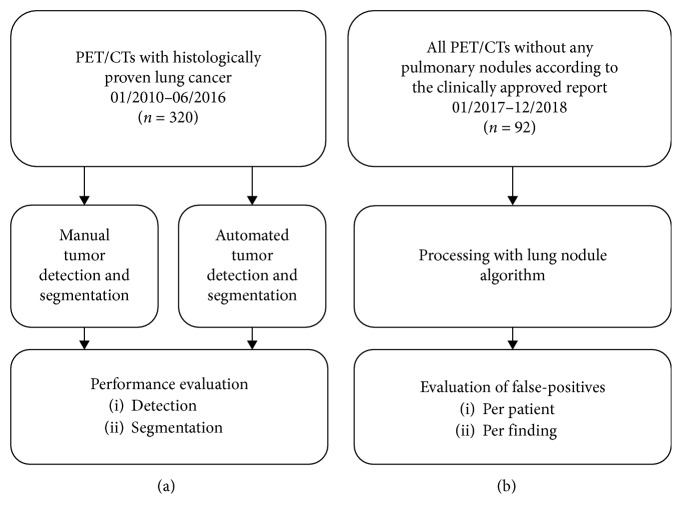
Study workflow for (a) lung tumor population and (b) nodule negative population.

We compiled two datasets using an in-house-developed Radiology Information System/Picture Archiving and Communication System (RIS/PACS) search engine: First, we retrospectively identified ^18^F-fluorodeoxyglucose- (FDG-) PET/CTs with histologically proven primary lung cancer that were acquired at our institution between 01/2010 and 06/2016. Selection criteria were protocol name, time period, and verified tumor histology according to our pathology archive. This resulted in 320 PET/CTs (lung tumor population). Second, for the creation of a dataset with exams not containing pulmonary nodules, appropriate PET/CTs were selected with the criteria protocol name, time period (01/2017–12/2018), and the presence of the text string “no pulmonary nodules” in the clinically approved reports. This resulted in 92 PET/CTs (nodule negative population). The study workflow is displayed in Figure 1.

### 2.2. Imaging Protocols

PET/CT examinations were performed on two integrated PET/CT systems: on a Discovery STE with 16-slice CT (GE Healthcare, Chalfont St Giles, UK) from 01/2008 to 11/2015 and on a Biograph mCT-X RT Pro Edition with 128-slice CT (Siemens Healthineers, Erlangen, Germany) from 12/2015 to 12/2016. Scans were obtained 1 hour after intravenous injection of 5 MBq FDG/kg body weight at glycemic levels below 10 mmol/L and previous fasting for at least 6 h. The CT component of the combined PET/CT examination was acquired with the following parameters: Discovery STE: slice thickness 3 mm, i50f kernel, X-ray tube voltage 120 kVp (SD: 0 kVp), exposure 80 mAs (SD: 15 mAs), CTDIvol 5.8 mGy (SD: 1.7 mGy), and DLP 536 mGy *∗* cm (SD: 100 mGy *∗* cm). Biograph mCT-X: slice thickness 3 mm, i50f kernel, X-ray tube voltage 120 kVp (SD: 0 kVp), 37 mAs (SD: 18 mAs), CTDIvol 3.1 mGy (SD: 1.5 mGy), and DLP 294 mGy *∗* cm (SD: 146 mGy *∗* cm). In 21 cases, Iopromide (Ultravist 370, Bayer Pharma, Germany, Berlin) was applied as contrast agent at a mean dose of 87.1 ml (SD: 24.9 ml). All other scans were acquired without contrast.

### 2.3. Ground Truth Segmentation

Manual tumor segmentations with reference to the clinically approved report were performed as previously described [10]. The PET/CT image dataset of each patient was segmented via a modified 3D-slicer-based segmentation tool (version 4.6.2, Slicer Python Interactor 2.7.11, Boston, USA). Segmentation of the data involved in this analysis was performed by a dual-board-certified radiologist and nuclear medicine physician with 10 years' experience in PET/CT reading (A. S., *n* = 137) as well as a radiology resident with 2 years of professional experience that was supervised by A. S. (T. W., *n* = 183). Tumors were segmented as a 3D volume defined by consecutive 2D regions of interest (ROIs) that were delineated on all transversal slices of the CT component showing a lesion. Fused PET information was used in addition whenever the tumor boundaries were not clearly definable on CT.

### 2.4. Algorithm Characteristics

The transversal 3 mm low-dose CT series of the PET/CTs with histologically proven primary lung tumor (*n* = 320) as well as the CT series of the PET/CTs negative for pulmonary nodules (*n* = 94) served as the only input for the in-house-deployed AI-based research algorithm for detection and segmentation of lung nodules. The image data were processed in three steps: First, lung and lung lobe segmentation was performed by a deep image-to-image network (DI2IN) that was trained on chest CTs acquired on scanners of multiple vendors. Its architecture has previously been described for liver segmentation by Yang et al. [24]. Second, nodule detection was performed by nodule candidate generation (NCG) and false-positive reduction (FPR). The NCG is a 3D region proposal network based on faster-RCNN [25] that outputs suspicious regions called “nodule candidates” and assigns probability scores. Then, for each nodule candidate, a small patch around it was sampled and sent to the FPR module consisting of several Res-Net units [26]. The FPR module further evaluated the likelihood for the nodule candidate to be a true nodule or a false positive by updating the scores generated by the NCG module. The final decision was made by taking the weighted sum of the scores generated by NCG and FPR modules. The training data for the nodule detection algorithm contained nodules up to a diameter of 3 cm. Third, nodules were segmented by an algorithm based on region growing. The principle of this method has been previously described by Hojjatoleslami and colleagues [27]. In the interest of improved readability, these three interlinked algorithms will be referred to as “algorithm” in this paper. None of the selected PET/CTs within the study was used to train the algorithm or to adapt hyperparameters.

### 2.5. Data Analysis

The output of the AI algorithm pipeline was the transversal chest CT component of the PET/CT with overlays for lung lobe boundaries and tumor boundaries of detected tumors. This output series also contained specifications of volume (Volume_AI_), 2D diameter, and location (lung lobe) for every detected tumor and served as the index test. The reference standard was the CT component of the PET/CT for detection and the volumes that were calculated from the 3D tumor masks that resulted from the manual image segmentation process (ground truth volumes: Volume_GT_). For each case, the segmented tumor was visually correlated with the output series of the algorithm and it was recorded whether the tumor was detected or not. The correctness of the indication of tumor location (lung lobe) was checked. We additionally established whether a lesion contacted parietal pleura or not by consensus reading (A. S. and T. W.). Finally, we reviewed the output series of the nodule negative population to describe numbers of and reasons for false-positive findings.

### 2.6. Statistical Analysis and Radiomics

Statistical analysis was performed using IBM SPSS Statistics for Windows, Version 22.0 (IBM Corp., Armonk, NY). Scatterplots and graphs were created with JMP, Version 14.2 (SAS Institute Inc., Cary, NC). For descriptive analyses of continuous data, we calculated the mean and standard deviations. To test for association between two or more categorical variables, we used the chi-squared test. To test for statistical differences among the means of two or more groups, we conducted a one-way analysis of variance. Normal distribution was assessed with the Shapiro–Wilk test, histograms, and Q-Q plots. To analyze the influence of histology, location, pleural contact, and maximal axial diameter on detection rates, we performed a binomial logistic regression with detection (yes/no) as the dependent variable. In this model, the largest histology subgroup and the most common location regarding the lung lobe (for location) were set as reference categories of the categorical variables. For the analysis of segmentation performance, all tumors with automatically calculated tumor volumes (Volume_AI_) were considered (=all tumors detected). We used the Pearson correlation coefficient to assess the relationship between Volume_GT_ and Volume_AI_. *p* values less than 0.05 were defined to indicate statistical significance.

To elucidate the influence of textual features on detection rates, we extracted 200 radiomic features with Pyradiomics version 2.1.0 [28]. Least absolute shrinkage selection operator (LASSO) regression and extended Bayesian information criterion (EBIC) were used for feature selection in Stata Statistical Software Release 15 (StataCorp, College Station, TX). Selected features were then transferred into a logistic regression model and the predictive power was assessed. Youden cutoff values were generated for each selected feature [29].

## 3. Results

### 3.1. Lung Tumor Population

#### 3.1.1. Population Characteristics

The mean patient age was 66.7 years (SD: 10.7 years). 70.3% of the patients were male (*n* = 225), and 29.7% were female (*n* = 95). The mean tumor volume was 68.2 cm^3^ (SD: 125.6 cm^3^; T1 = 3.0 cm^3^, T2 = 17.8 cm^3^, T3 = 56.7 cm^3^, and T4 = 210.0 cm^3^), and the mean axial tumor diameter was 5.0 cm (SD: 3.4 cm). Tumors were located in all lobes (right upper lobe: *n* = 101; middle lobe: *n* = 19; right lower lobe: *n* = 50; left upper lobe: *n* = 88; left lower lobe: *n* = 62). All T-categories were represented in the dataset with the following distribution: T1: *n* = 83; T2: *n* = 106; T3: *n* = 51; T4: *n* = 80. There were no statistically significant differences between the patients included in the T-categories regarding age and gender (*χ*^2^ = 1.217, *p*=0.749). The distribution of tumor histology is shown in Table 1.

#### 3.1.2. Detection

**Figure 2:**
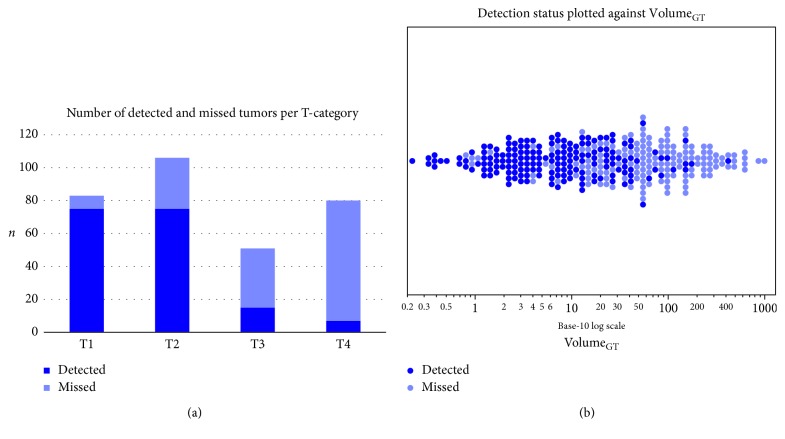
Tumors and their detection status. Tumors detected by the algorithm are visualized in dark blue and missed tumors in light blue. (a) Histogram per T-category. (b) Detection of tumors depending on the ground truth volumes. Every dot represents one tumor. *X*-axis with Volume_GT_ in cm^3^, in base-10 log scale.

The attribution of a lesion to the corresponding lung lobe was correct in 100% of the detected lesions. Detection rates differed significantly across T-categories and declined towards advanced tumors: 90.4% for T1 (75 of 83), 70.8% for T2 (75 of 106), 29.4% for T3 (15 of 51), and 8.8% for T4 (7 of 80). This detection decline is also reflected in Figure 2(a) that shows the number of detected and missed tumors by T-category and Figure 2(b) that displays detection of tumors depending on the ground truth volume. Furthermore, mean Volume_GT_ was smaller for detected lesions (18.6 cm^3^; SD: 39.3 cm^3^) as compared to missed lesions (125.9 cm^3^; SD: 161.8 cm^3^).

Binominal logistic regression conducted to explore factors that influence detection rates showed that tumors with a larger maximal axial diameter and tumors with pleural contact were more likely to be missed by the detection algorithm (both *p* < 0.001). The results of this analysis are summarized in Table 2. Interestingly, squamous cell carcinomas and SCLC had a slightly higher likelihood to be missed compared to adenocarcinomas (*p* < 0.001 and *p*=0.015, respectively). Location of a lesion in a specific lung lobe did not influence detection rates. With an Exp(B) of 74.4, pleural contact was by far the most relevant factor for nondetection in the model. This is also reflected by the fact that 94 of 95 lesions without pleural contact were detected (98.9%), while only 78 of 225 lesions with pleural contact were correctly identified (34.7%).

Table 3 summarizes the results of the radiomic analysis. It revealed that first order, shape, and texture features were significantly different in detected and missed tumors (*p* < 0.001). Tumors with finer, less heterogeneous texture (e.g., CT_glrlm_GrayLevelNonUniformityN: Lasso coefficient = −1.0776312, Youden cutoff = 0.1166608) and rounder shape (e.g., shape_Sphericity: Lasso coefficient = 0.2268932, Youden cutoff = 0.4293948) were more likely to be detected by the algorithm. Interestingly, three PET features (PET_firstorder_10Percentile, PET_firstorder_Maximum, PET_gldm_DependenceEntropy) indicated whether or not a tumor is detected on the CT component.

#### 3.1.3. Segmentation

**Figure 3:**
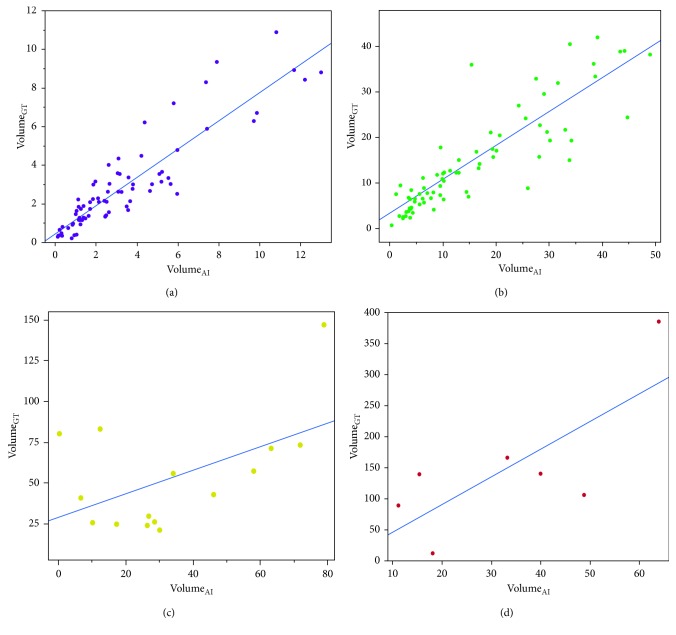
Segmented ground truth volumes (Volume_GT_) in cm^3^ (*Y*-axis) plotted against automatically calculated volumes (Volume_AI_) in cm^3^ (*X* axis) with linear regression line for (a) T1, (b) T2, (c) T3, and (d) T4.

**Figure 4:**
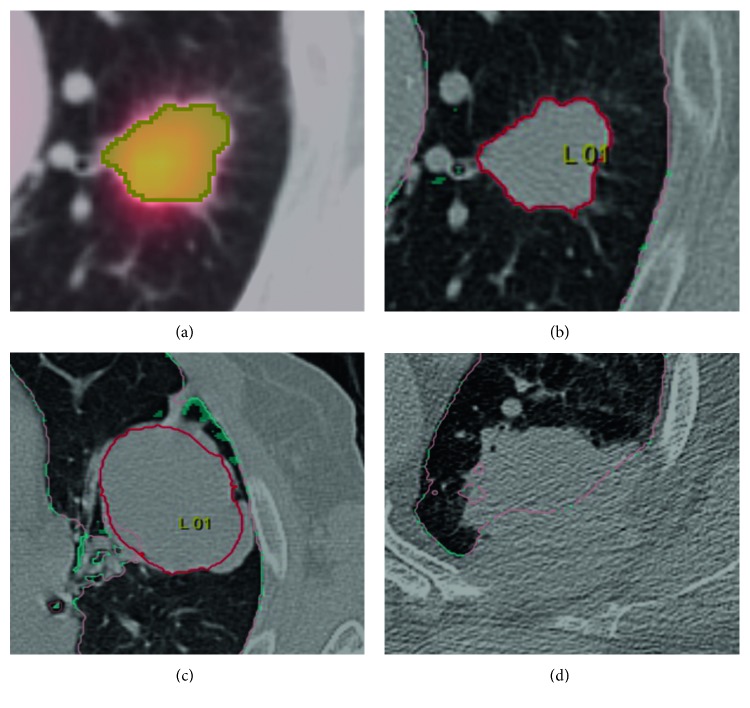
Examples for (a) manual segmentation of a T1 tumor without pleural contact with (b) corresponding excellent segmentation by the algorithm, (c) an incompletely segmented T3 lesion with pleural attachment, and (d) a completely missed T4 lesion with infiltration of the chest wall.

All tumors detected by the algorithm were included in the second step of our analysis that investigated the segmentation performance (all: *n* = 172; T1: *n* = 75; T2: *n* = 75; T3: *n* = 15; T4: *n* = 7). We found a positive correlation between volumes calculated by the algorithm and ground truth volumes (Pearson correlation coefficient: *r* = 0.634, *p* < 0.001). As for detection rates, there were differences regarding T-categories: *r* = 0.908 for T1 (*p* < 0.001), *r* = 0.797 for T2 (*p* < 0.001), *r* = 0.520 for T3 (*p*=0.047), and *r* = 0.748 for T4 (*p*=0.053). This correlation is displayed in Figures 3(a)–3(d). It is worth mentioning that due to the low detection rate only seven T4 tumors were included and therefore the high Pearson correlation coefficient is likely related to random effects. Automatically calculated volumes of tumors that had no contact to pleura had a stronger correlation with ground truth volumes (*r* = 0.971, *p* < 0.001) as compared to tumors with pleural contact (*r* = 0.586, *p* < 0.001) for all T-categories. The volumes of larger tumors were systematically underestimated by the algorithm.  Figure 4 displays a typical example of a T1 lesion without pleural contact that was manually segmented (a) as well as correctly segmented by the algorithm (b).  Figure 4(c) shows an incompletely segmented T3 lesion with pleural attachment, and Figure 4(d) illustrates an invasive, completely missed T4 lesion.

### 3.2. Nodule Negative Population

Mean age of the patients was 63.2 years (SD: 16.6 years). There were 60.6% males (*n* = 57) and 39.4% females (*n* = 37). There were 39 false-positive findings (FP). This corresponds to 0.41 FP per patient. FPs were caused by dystelectases (*n* = 18), intrapulmonary vessels (*n* = 12), hilar calcified lymph nodes (*n* = 3), detection of ribs (*n* = 2), and a breathing artifact (*n* = 1).

## 4. Discussion

The evaluated AI-driven algorithm allows for excellent detection and segmentation of pulmonary T1 lesions (detection rate: 90.4%; excellent correlation of Volume_AI_ and Volume_GT_: *r* = 0.91) and good detection and segmentation of T2 tumors (detection rate: 70.8%; correlation of Volume_AI_ and Volume_GT_: *r* = 0.80) on the CT component of PET/CTs. Given the fact that the algorithm is designed for the detection of lung nodules smaller than 3 cm, such good performance on tumors with a diameter of up to 5 cm is remarkable. This is even truer considering the fact that the CT series used as input for the algorithm had a slice thickness of 3 mm and were acquired in free breathing and mostly nonenhanced technique. In more advanced tumors (T3/T4), detection and segmentation are more challenging and subsequently detection rates are low. Furthermore, the segmentation mask volumes for T3/T4 tumors systematically underestimate ground truth volumes. It is therefore an important finding that the tested CAD system has conceptional limitations concerning the detection of advanced lung tumors, and human inspection is still necessary in these cases.

The first step of CAD systems is to detect the location of lesions in medical images [30]. Most previous studies used CT datasets from lung cancer screening trials (e.g., NLST) with nodule size between 3 and 30 mm [19]. As an exception, Dandil et al. analyzed 52 malignant and 76 benign lesions with a size range from 4 to 58 mm, but only 12.5% of these nodules were bigger than 20 mm in diameter [20]. They reported a sensitivity of 92.3%, which is in line with the detection performance we found for the comparable group of T1 tumors. Earlier this year, Vassallo et al. compared unassisted and cloud-based CAD of pulmonary nodules in patients with extrathoracic malignancy [13]. A total of 215 lung nodules with a diameter between 3 and 28 mm in 75 patients were used for evaluation. Stand-alone CAD sensitivity was 85%, and the mean false-positive rate per scan was 3.8. These performance measures are representative for recently published studies on lung nodule CAD software [12, 14–18, 21]. Our results show a sensitivity of 90.4% for small tumors with a diameter of up to 30 mm with a far superior rate of false-positive findings per exam of 0.41 on the nodule negative population. This low rate of false-positive findings is a prerequisite for integration into existing clinical workflows and acceptance by radiologists and nuclear medicine physicians. Liang et al. tested four CAD systems at two time points for the detection of nodules with a mean diameter of 4 mm and 11 mm, respectively, and found sensitivities ranging from 52% to 82% [11]. Again, false-positive rates of 0.6–7.4 per exam ranged above the ones we found and —in line with our results—were often caused by detection of blood vessels and bone. They did not identify dystelectasis as a reason for FP findings—the most frequent cause we found. This can be explained by the fact that we tested on PET/CTs acquired in free breathing technique, while Liang and colleagues evaluated on chest CTs acquired in deep-inspiration breath-hold technique [11]. Of interest and with only one exception, they as well as some other authors [31, 32] reported higher detection rates of the CADs for isolated cancers as compared to those attached to the pleura. This supports our finding that pleural contact negatively affects detection. It is important to understand that these features are not totally independent from each other. For example, advanced tumors more likely invade structures adjacent to the lung, which means that pleural contact exists. Of interest, we found no dependency of lesion detection on the location within the lung, whereas Liang and colleagues reported a higher probability of detection for nodules in lower lobes for three of the four evaluated CAD systems [11]. However, the effect was small and not statistically significant.

Our radiomics analysis revealed further features that influence the detection rates: a finer, less heterogeneous and rounder texture was associated with better detection. While the utility of texture analysis for the differentiation of benign vs. malign lung lesions [33, 34], the differentiation of histologic subtypes [35, 36] and the prediction of progression [37–39] is well established, more studies on its influence on detection rates are warranted. Regarding tumor histology, our analysis revealed slightly lower detection rates for SCLC and squamous cell carcinomas as compared to adenocarcinomas. Due to the low number of cases in the two groups, however, these results are likely to be influenced by random effects. Another explanation could be that no preliminary stages of adenocarcinoma were included in our patient population. It is well known that adenocarcinoma with lepidic growth pattern has lower detection rates by human readers [40].

After detection, segmentation of lung lesions is the subsequent step that, if done correctly, paves the way to a plethora of secondary analyses that are currently developed within the context of AI, radiomics, and personalized medicine. In this context, Owens et al. compared contours of 10 lung tumors ranging from 1.1 cm^3^ to 10.5 cm^3^ defined by human readers in consensus, corresponding to our categories T1 and T2, with 2 semiautomatic segmentation methods: Lesion Sizing Toolkit (LSTK) and GrowCut [41]. For these semiautomatic tools, the mean Dice similarity coefficients were 0.88 ± 0.06 and 0.88 ± 0.08 for LSTK and GrowCut, respectively, indicating very good segmentation quality. Our results which reveal an excellent correlation of Volume_GT_ and Volume_AI_ for T1 (*r* = 0.90) and a good correlation for T2 tumors (*r* = 0.70) are in line with these findings. Various other studies assessed automated segmentation methods for the segmentation of lung nodules on the Lung Image Database Consortium-Image Database Resource Initiative (LIDC-IDRI) dataset (diameters: 2 mm–38 mm, again corresponding to T1 and T2-category of our dataset) and reported overlaps of ground truth and automatically generated segmentation masks of 50.7% [42], 58% [43], 63% [31], 69%, and 71.2% [44], respectively. Furthermore, Hassani et al. mention in their review that difficulties of semi automated and fully automated systems in segmenting subpleural nodules are due to masking of margins by adjacent normal structures [45]. Our results confirm this finding, showing a much better correlation of Volume_GT_ and Volume_AI_ for isolated lesions (*r* = 0.97) as compared to attached lesions (*r* = 0.59).

According to current guidelines, FDG-PET/CT is considered the standard imaging procedure of choice for noninvasive staging of lung cancer [5]. The CT component of this examination is often acquired in free breathing using thicker slices (3 mm) and a lower dose compared to diagnostic chest CTs. In opposition to Marten et al., who reported significantly dropping detection rates for increasing reconstruction slice thicknesses (0.75 mm: 73.9%, 2 mm: 59.0%, 4 mm: 4.4%) [46], we found detection rates for the comparable T1-category collective that are equal or superior to those reported by other authors for 1 mm slice thickness. This can be explained by the fact that detection rates of DCNN detection algorithms used in our study are superior compared to techniques based on histogram analysis and thresholding used years ago. Teramoto et al. evaluated a CAD system that used both the CT and PET component to generate candidate lesions with a subsequent reduction of false-positive findings through a convolutional neural network (slice thickness: 2 mm; 104 cases with 183 nodules) [22]. They report a sensitivity regarding detection of 91% that is very similar to the one we found but a higher rate of false-positive findings per case (4.9). An inclusion of the information contained in the PET-component of the FDG-PET/CT could be a direction of further development of the CAD we tested.

There are several limitations of our work. First, manual segmentation was performed by two readers in random order without consensus or double reading. Both, consensus and double reading are time-consuming tasks and therefore not practicable in this study with a total of 320 lesions. Second, the assessment of segmentation quality was based on comparison of the automatically calculated tumor volumes with ground truth volumes. More advanced methods like Dice similarity coefficients or Hausdorff distances could not be applied since space coordinates were not accessible in the manually created tumor masks. Third, for the creation of manual tumor masks, the FDG-PET component was considered whenever tumor borders could not be well delineated on the CT component, while automated tumor detection was performed only on the CT component. Inclusion of the information contained in the PET components could possibly increase detection rates and segmentation quality. Fourth, the analysis was conducted in two steps: detection and segmentation. Due to lower detection rates for more advanced tumors, a selection bias in step two of the analysis could positively influence segmentation performance in this group.

In conclusion, the tested algorithm facilitates a fast and reliable detection and 3D segmentation of pulmonary T1 and T2 tumors that also works well on the CT component of PET/CTs acquired in free breathing and with a slice thickness of 3 mm. The detection and segmentation of more advanced lung tumors is currently imprecise due to the conception of the algorithm for lung nodules. Consequently, there is still an unmet need for CAD applications that also cope with the more complex segmentation tasks required in the context of lung cancer staging. Future efforts must therefore focus on this collective to facilitate segmentation of all tumor types and sizes and bridge the gap between CAD applications for screening and staging of lung cancer.

## Figures and Tables

**Table 1:** Distribution of the lung tumor histology subtypes.

Tumor histology	*n*	%
Adenocarcinoma (AC)	174	54.2
Squamous cell carcinoma (SCC)	79	24.6
NSCLC not specified (NOS)	25	7.8
SCLC	15	4.7
Other^*∗*^	28	8.7

^*∗*^Large cell carcinoma, neuroendocrine tumor (NET), sarcomatoid carcinoma, spindle cell carcinoma, typical carcinoid, and combined carcinomas (NET + SCLC; SCLC + SCC; NET + SCC; NET + AC).

**Table 2:** Results of the binomial logistic regression.

Independent variables	*p*	Exp(*B*) with 95% CI
Histology subtype		
Reference: adenocarcinoma		
(1) Squamous cell carcinoma	**<0.001**	**0.209 (0.089–0.490)**
(2) NSCLC (NOS)	0.181	0.443 (0.134–1.461)
(3) SCLC	**0.015**	**0.093 (0.014–0.636)**
(4) Others	0.653	0.765 (0.237–2.464)
Location (lobes)		
Reference: right upper lobe		
(1) Middle lobe	0.350	0.499 (0.116–2.145)
(2) Right lower lobe	0.495	1.446 (0.502–4.167)
(3) Left upper lobe	0.905	1.054 (0.448–2.480)
(4) Left lower lobe	0.902	0.943 (0.369–2.408)
Pleural contact	**<0.001**	**74.400 (9.345–592.324)**
Maximal axial diameter	**<0.001**	**0.953 (0.938–0.969)**

Detection (yes/no) was set as dependent variable. Independent variables: histology (categorial), location (categorial), pleural contact (dichotomous), and maximal axial diameter (continuous). Exp(*B*) is the exponentiation of the *B* coefficient.

**Table 3:** Results of the radiomic analysis with features from Pyradiomics.

Selected feature	Lasso coefficient	Youden cutoff
CT_glrlm_GrayLevelNonUniformityN	−1.0776312	0.1166608
PET_firstorder_10Percentile	−0.0344698	1.7492108
PET_firstorder_Maximum	−0.0022762	6.9905767
PET_gldm_DependenceEntropy	0.0716689	2.2174546
shape_Maximum2DdiameterSlice	−0.0043233	32.866422
shape_Sphericity	0.2268932	0.4293948

## Data Availability

The volumetric data are all published within this manuscript. A large part of the data are patient data and thus confidential. Upon request, a minimal anonymized dataset will be available to interested researchers.
